# Organic and Inorganic Carbon in Paddy Soil as Evaluated by Mid-Infrared Photoacoustic Spectroscopy

**DOI:** 10.1371/journal.pone.0043368

**Published:** 2012-08-17

**Authors:** Du Changwen, Zhou Jianmin, Keith W. Goyne

**Affiliations:** 1 The State Key Laboratory of Soil and Sustainable Agriculture, Institute of Soil Science Chinese Academy of Sciences, Nanjing, People’s Republic of China; 2 Department of Soil, Environmental and Atmospheric Sciences, University of Missouri, Columbia, Missouri, United States of America; Consejo Superior de Investigaciones Cientificas, Spain

## Abstract

Paddy soils are classified as wetlands which play a vital role in climatic change and food production. Soil carbon (C), especially soil organic C (SOC), in paddy soils has been received considerable attention as of recent. However, considerably less attention has been given to soil inorganic carbon (SIC) in paddy soils and the relationship between SOC and SIC at interface between soil and the atmosphere. The objective of this research was to investigate the utility of applying Fourier transform mid-infrared photoacoustic spectroscopy (FTIR-PAS) to explore SOC and SIC present near the surface (0–10 µm) of paddy soils. The FTIR-PAS spectra revealed an unique absorption region in the wavenumber range of 1,350–1,500 cm^−1^ that was dominated by C-O (carbonate) and C-H bending vibrations (organic materials), and these vibrations were used to represented SIC and SOC, respectively. A circular distribution between SIC and SOC on the surface of paddy soils was determined using principal component analysis (PCA), and the distribution showed no significant relationship with the age of paddy soil. However, SIC and SOC were negatively correlated, and higher SIC content was observed near the soil surface. This relationship suggests that SIC in soil surface plays important roles in the soil C dynamics.

## Introduction

Surface horizon soils are primarily composed of particles of unbound organic matter and soil aggregates covered by an organic layer, and this organic layer holds some inorganic components (such as metals and carbonates) within the matrix [1], [2]. These organic materials comprise the stock of soil organic carbon (SOC), whereas carbonates primarily represent the stock of soil inorganic carbon (SIC) [3]. Through dynamic processes, there is a relationship between soil carbon (C) and atmosphere C; therefore, soil can partly buffer rising atmospheric CO_2_ concentrations [4]. Indeed, the pool of C in soil and vegetation is approximately three times higher than in the atmosphere indicating that any increase of C sequestration by soils could significantly offset the rising of atmospheric CO_2_ and the resulting in global warming [5], [6]. Moreover, elevated CO_2_ often stimulates primary production and as a feedback, greater C input is expected to increase C sequestration in soil. Soil organic C, such as humic materials, is relatively stable, but organic C at the outer edge of soil particles and aggregates is more dynamic since it is greatly influenced by water, atmosphere, and biota [7]. Although C at the edge of particles and aggregates occupies a small percentage of total SOC, it is the most active part of soil C and it plays important roles in the soil C fluxes as well as biogeochemical reactions [8], [9].

Paddy soils are the largest anthropogenic wetlands on earth, but they are highly modified by anthropogenic activities [10]. Due to their wide extent, paddy soils not only provide food for millions of people but they also play a role in climatic change through fluxes of carbon dioxide (CO_2_) and methane [11]–[13]. Paddy soil research has focused primarily on SOC [14]–[16], but much less attention has been devoted to SIC even though it also contributes to soil C stocks [17], [18]. One of the main reasons that the relationship between SOC and SIC remains understudied is due to the difficulty of differentiating SOC and SIC with conventional extractant-based chemical methods [2], [19].

Fourier transform mid-infrared photoacoustic spectroscopy (FTIR-PAS) and has been applied in a wide disciplines [20] and it offers an alternative option for studying SOC and SIC in soils. This novel technique has recently been applied to study soils, and these studies have demonstrated that FTIR-PAS is very suitable for investigating soil samples with varied morphology and particle size [21]–[23]. One promising feature associated with FTIR-PAS is its depth profiling function [24], [25], which permits investigating chemical characteristics at differing depths from the surface of soil particles and aggregates via collection of spectra with different moving mirror velocities. The objectives of this study were to investigate SOC and SIC on the outer surface of paddy soil particles and aggregates using the depth profiling technique of FTIR-PAS and to investigate relationships between SOC and SIC in paddy soils.

## Materials and Methods

### Paddy Soil Samples

Contemporary paddy soil samples, a total of 739, were collected from eastern China (119.3120–121.0908 E, 30.7955–32.0471 N), and additional 117 historic paddy soil samples were collected from an archaeological site in the city of Cixi also located in eastern China (121.2333 E, 30.1667 N). Contemporary paddy soils were sampled at all sites from 0–15 cm depth. The age of ancient paddy soils ranging from 50 to 2,000 years old was determined by ^14^C dating in the organic matter and in the carbonized rice [11]. All soil samples were air-dried, passed through a 2 mm sieve, and kept refrigerated at 4°C prior to use.

### FTIR-PAS Analysis

Paddy soil sample spectra were collected using a Nicolet 380 spectrophotometer (Thermo Electron, USA) equipped with a photoacoustic cell (Model 300, MTEC, USA). Samples (∼ 200 mg) were placed in the cell holding cup (5 mm diameter×3 mm height), after which the cell was purged with dry helium (20 mL min^−1^) for 30 seconds to minimize interferences due to water vapor and impurities. The samples were then rapid scanned from 500 to 4,000 cm^−1^ wavenumber with a resolution of 4 cm^−1^ and moving mirror velocity of 0.64 cm s^−1^. Following these analyses, 118 paddy soil samples (119.3120–119.4167E, 31.5220–32.0353) randomly selected from 739 contemporary paddy soil samples were scanned at moving mirror velocities of 0.16, 0.32, 0.64 and 1.89 cm s^−1^. All FTIR-PAS spectra were normalized to a carbon black background, and 32 successive scans were recorded and standardized for each sample.

The profiling depths associated with each FTIR-PAS were calculated using the following function [26]:
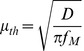
(1)where *µ_th_* is thermal diffusion length (µm), *D* is soil thermal diffusion coefficient (cm^2^ s^−1^), *f_M_* is modulated frequency of infrared incident (Hz), which equals moving mirror velocity plus wavenumber in rapid FTIR-PAS scanning. A value of *D
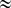
*10^−3^ cm^2^ s^−1^ from reference [27] was chosen for organic materials.

### Processing of Spectra Data

The spectra were pre-processed by applying a smoothing filter and normalizing the amplitudes [22]. Data reduction was achieved through use of principal component analysis (PCA), which is commonly used to reduce the dimensionality of infrared spectra and yields a small number of coefficients (so called PCA scores) that retain most of the variability (information) present in the original data.

Modeling of the relationship between soil FTIR-PAS and soil age was performed using partial least square regressions (PLSR) [28]. For PLSR modeling, 87 samples and 30 samples were used in the calibration and validation sets, respectively. The PLS factor was optimized as six [23] and root mean square error (RMSE) was calculated to evaluate model performance. Software of Matlab was used in the processing of spectral data.

## Results

### Characterization of FTIR-PAS Spectra of Paddy Soils

**Figure 1:**
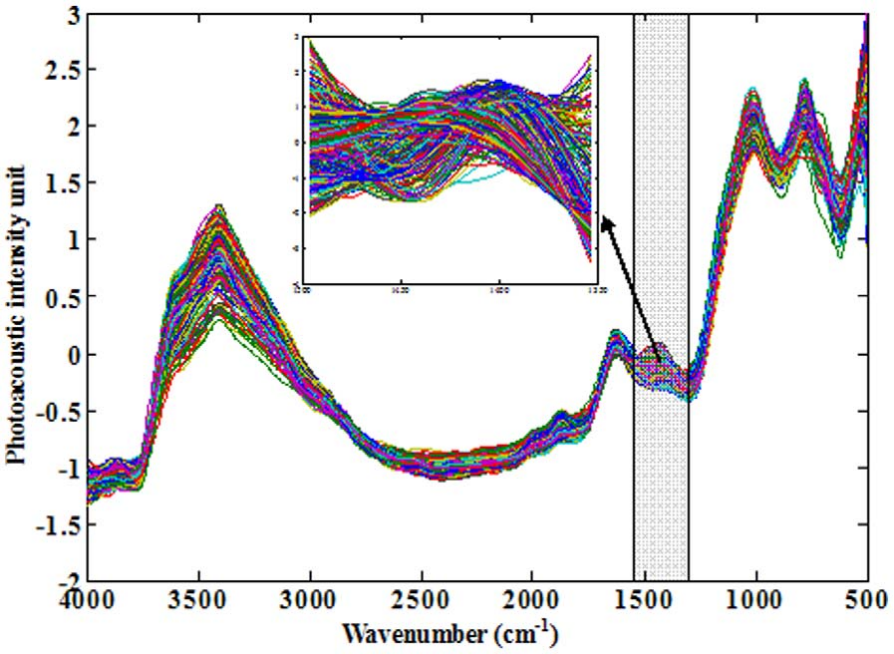
FTIR-PAS spectra of paddy soils (*n* = 739) collected with a moving mirror velocity of 0.64 cm s^−1^. The shaded frame showed the absorption band in the wavenumber region of 1,350–1,500 cm^−1^.

Figure 1 shows the spectral characteristics of paddy soils from eastern China (*n* = 739). Generally, three main absorption regions are observed in each spectrum: (1) 2,000–4,000 cm^−1^; (2) 1,200–2,000 cm^−1^; (3) and 500–1,200 cm^−1^. The 2,000–4,000 cm^−1^ region contains O-H, N-H and C-H vibrations from almost all soil components (i.e., all organic materials and clay minerals), thus the absorption band is relatively broad. For the absorption region of 500–1,200 cm^−1^, usually called as fingerprint region, two strong absorption bands are present and they represent stretching and bending vibrations from various groups (e.g., C-O, C-H, Si-O-Si, etc.). Strong interferences were also observed in this range [29]. Therefore, the absorption region of 1,200–2,000 cm^−1^ appears to be the candidate region for SOC/SIC related soil analysis. Vibration bands at ∼1,600 cm^−1^ and ∼1,100 cm^−1^ are primarily associated with soil water and silicon contained within clay minerals, respectively [21]. However, there is relatively little interference in the absorption region of 1,350–1,500 cm^−1^. The main vibrations in this region are C-H bending vibrations from organic materials (including aliphatic and aromatic C-H) (1,300–1,500 cm^−1^) and C-O stretching vibrations from carbonates (1,400–1,500 cm^−1^) [29]–[31].

**Figure 2:**
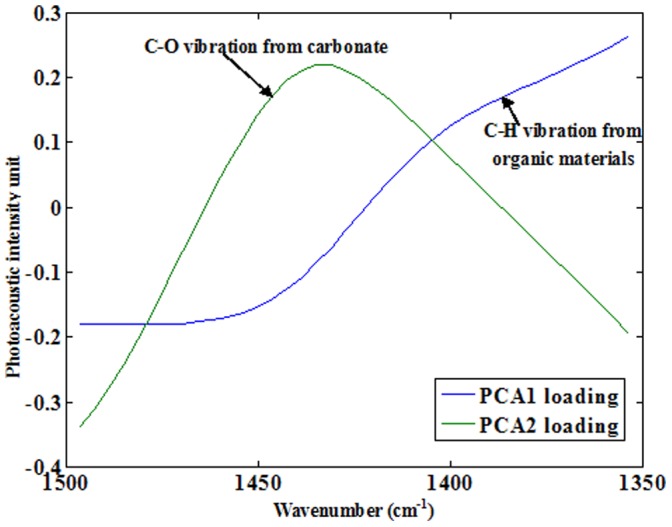
The first two PCA loadings in the wavenumber region of 1,350–1,500 cm^−1^ for contemporary paddy soil samples (*n* = 739). The explained variances a PCA1 and PCA2 were 57.5% and 30.2%, respectively.

A principal component analysis (PCA) was conducted using data extracted from the FTIR-PAS spectra of the contemporary paddy soils. The analysis indicated two main principle components, PCA1 and PCA2, could explain variances of 57.5% and 30.2%, PCA1 and PCA2, respectively. The loadings associated with PCA1 and PCA2 in the candidate region are shown in Figure 2. The loading of PCA1 shows characteristics associated with C-H bending vibrations over a relatively wide wavenumber range (1,350–1,500 cm^−1^), and the loading of PCA2 shows the typical carbonate absorption band at ∼1,450 cm^−1^. The variance explained by the first two principle components was 87.7%, and the PCA loading spectra show very good separation of C-H from organic materials and C-O from carbonate in this spectral range. Thus, PCA1 and PCA2 were chosen to represent SOC and SIC, respectively.

### Relationship between SOC and SIC in Surface Layer of Spatial Contemporary Paddy Soils

**Figure 3:**
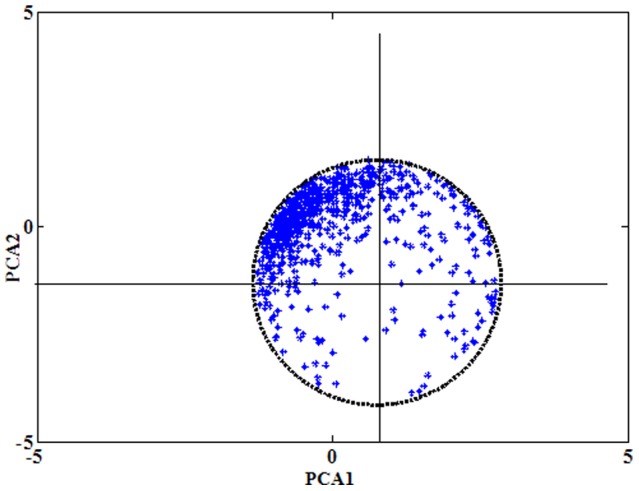
Circular distribution of the first and the second principal components associated with contemporary paddy soil FTIR-PAS spectra (*n* = 739) in wavenumber region of 1,350–1,500 cm^−1^. Spectra were collected with moving mirror velocity of 0.64 cm s^−1^. Thickness of the scanning depth was calculated as 6.1 µm; *r*, defined as soil carbon capacity unit.

PCA distribution of FTIR-PAS spectra of contemporary paddy samples collected with a moving mirror velocity of 0.64 cm s^−1^ were drawn over the wavenumber range of (1,350–1,500 cm^−1^). The distribution between SOC (PCA1) and SIC (PCA2), shown in Figure 3, exhibits a circular distribution with radius unit of 2.0 and original points of (0.8,−1.5).

**Figure 4:**
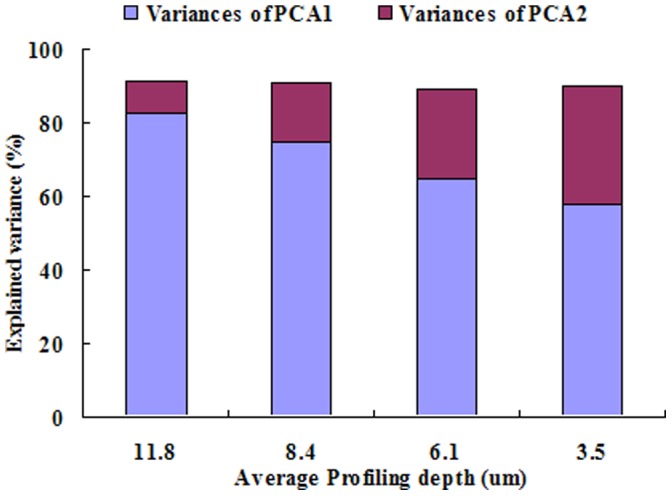
Variance explained by the first two PCA components (PCA1 and PCA2) in samples (*n* = 118) scanned at different profiling depths using FTIR-PAS. Four velocities of the moving mirror (0.16 cm s^−1^, 0.32 cm s^−1^, 0.64 cm s^−1^, 1.89 cm s^−1^) were used to perform depth profiling of soil samples in the wavenumber region of 1,350–1,550 cm^−1^. The corresponding average profiling depths were calculated as 11.8 µm, 8.4 µm, 6.1 µm, 3.5 µm, respectively.

From the 739 contemporary paddy soils, 118 samples were randomly selected for FTIR-PAS analysis at different profiling depths (3.5–11.8 um) with varied moving mirror velocities. A principle components analysis was conducted using data extracted from the wavenumber region of 1,350–1,500 cm^−1^ (Figure 4) to investigate the relationship between SOC and SIC as a function of profiling depth. For the profiling FTIR-PAS spectra, there were two main principal components, PCA1 and PCA2, which explained 90% of in the spectral range investigated, thus suggesting that interference from other vibrational bands was relatively low (about 10%). Furthermore, the explained variance of PCA2, increased with decreasing profiling depth, whereas explained variance associated with PCA1 showed the exact opposite trend with profiling depth.

**Figure 5:**
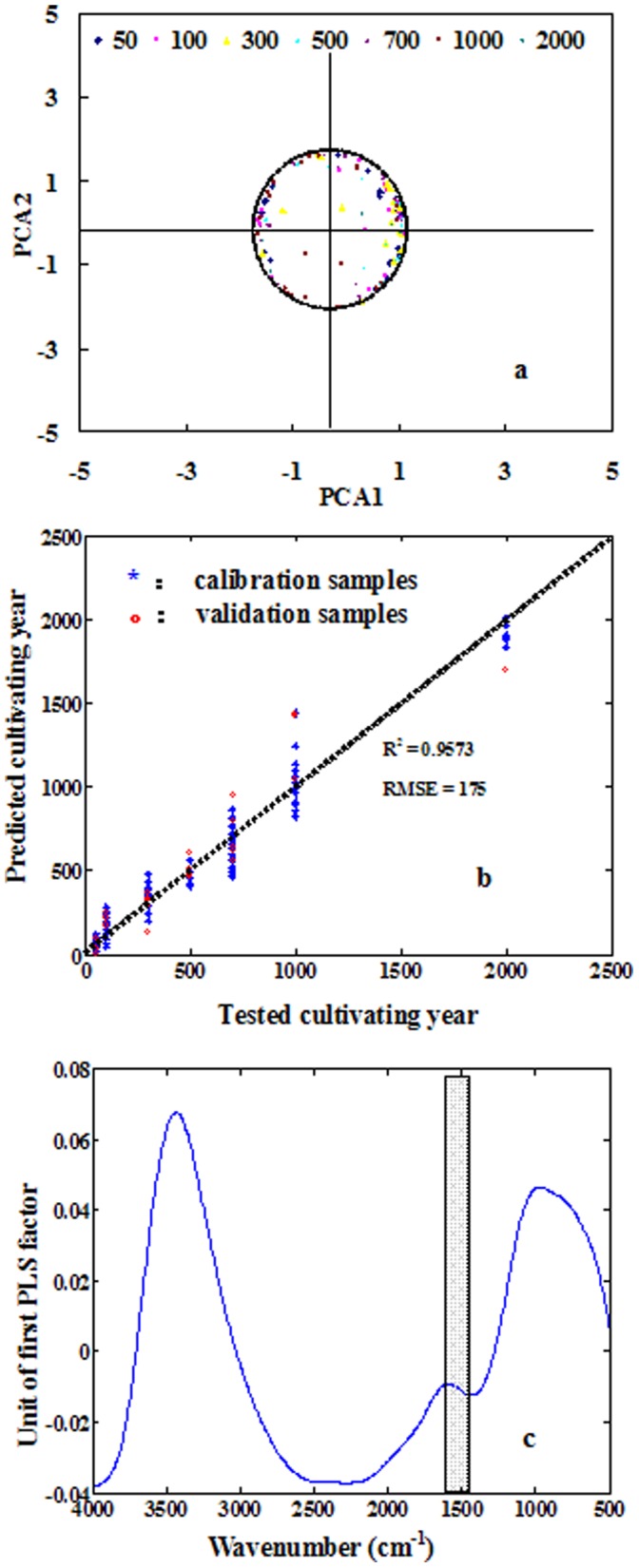
Analysis of FTIR-PAS spectra of historic paddy soils of different age (50–2,000) years using PCA and partial least square analysis (PLS) (*n* = 117): (a) distribution of the first (representing SOC) and the second (representing SIC) principal components in the wavenumber region of 1,350–1,500 cm^−1^; (b) the relationship between tested soil age and predicted soil age using PLSR model (87 soil samples were used in calibration and 30 soil samples were used in validation); (c), the PLSR loading spectra of first PLSR factor, which shows the main wavenumber region (1,350–1,500 cm^−1^) related to soil age (shaded frame). The moving mirror velocity during time of collection was 0.64 cm s^−1^.

The distribution of SOC and SIC demonstrated as a circle function in the spatial paddy soils, does it work for time series based archaeological soil samples? Temporal ancient paddy soil samples were used to check the C dynamic in surface soil. The occupied variances in the PCA1 and the PCA2 were 57.7% and 30.1%, respectively (very similar to the results of contemporary paddy soils); also, a circular distribution was observed with radius unit of 1.2 and original points of (−0.2,−1.5) (Figure 5a). There was also significant difference in the distribution density between the spatial contemporary paddy soils and temporal ancient paddy soil samples.

## Discussion

### Modeling of SOC and SIC in Surface Layer of Contemporary Paddy Soils

Using Eq. (1), the profiling depths associated with FTIR-PAS analysis of contemporary paddy samples collected with a moving mirror velocity of 0.64 cm s^−1^ were calculated to be 5.8 to 6.1 µm over the wavenumber range of (1,350–1,500 cm^−1^). The distribution between SOC (PCA1) and SIC (PCA2), shown in Figure 4, exhibits a circular distribution, which can be expressed with the following function:
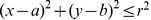
(2)where *x* (PCA1) denotes SOC, *y* (PCA2) denotes SIC, and *a*, *b*, *r* are constants. We assume that *a*, *b*, *r* are soil type dependent constants, where *a* and *b* might represent soil basic properties, and *r* represents soil C capacity unit. If the distribution circle is shifted to the origin of the graph (0, 0), the expression is changed to:



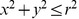
(3)From Figure 2, the distribution density is observed to be significantly different within the circle. The highest density of points is located in upper left-hand corner, near the border of the distribution circle; thus, in most of cases, the expression can be represented as:
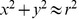
4


Assuming that *r* in Eq. (3) represents maximum soil C content, it can be deduced that *x* (SOC) or *y* (SIC) is less than *r*, indicating that the there would be a maximum SOC or SIC content in a specific soil type. It has been observed previously that SOC does not increase continuously even under conditions of great C input [32]. This was also verified in a long-term manure fertilization experiment conducted on a Fluvo-aquic soil [33]. Soil organic carbon increased during the first five to eight years, but thereafter a balance was reached.

From Eq. (2), neither a positive nor negative relationship can be drawn between SIC and SOC in the surface layer of soil particles and aggregates. However, a negative relationship between SOC and SIC can be deduced from Equation (4). This suggests that SOC content could possibly be limited by SIC content when soil C maxima has been reached for a specific soil. The apparent relationship between SIC and SOC could be important for global climatic change, as it may be possible for increases in SIC to compensate for losses in SOC in arable soils.

Differences in explained variances as a function of scanning depth (Figure 4) are likely due to differences in soil conditions favoring SIC accumulation at the edge of particles and aggregates. These result suggested that the SIC decreased with the profiling depth, while SOC increased with the profiling depth, and a negative relationship between SIC and SOC in this surface layer was observed. Calcite precipitation and dissolution in soils is dependent on a number of environmental and geologic factors, including temperature, concentrations of dissolved Ca^2+^ and CO_2_ in the soil pores, and the alkalinity and pH of the soil solution [17]. In general, the precipitation of calcite (as well as other carbonates) in soils is favored as Ca^2+^ concentration increases, CO_2_ concentration decreases, and pH and alkalinity are increased. Presumably, CO_2_ concentration is greater within organic particles and aggregates, due to diffusion limitations, than soil on or near the surface of particles which is in greater contact with the atmosphere or pores exchanging gases with the atmosphere. Lower CO_2_ concentrations at the surface of particles and aggregates as well as greater concentrations of cations, such as Ca^2+^, Mg^2+^, could result in increased carbonate mineral formation and precipitation [17], thus resulting in a greater apparent signal from SIC at more shallow scanning depths (e.g., 3.5 µm).

### Comparison of SOC and SIC in Contemporary and Historic Paddy Soils

Distribution density for the ancient paddy soil samples was almost entirely along the circle border, thus they were well fitted by Equation (4) and SIC was most likely negatively related with SOC. Because of the significantly larger *r* value (2.0 versus 1.2), the contemporary paddy soils might demonstrate a larger soil C stock than historic paddy soils, thus the paddy soil has a tendency to stock more C after longer cultivating time, which will contribute to restrain CO_2_ in the atmosphere. From this view, paddy soil showed a sustainable capability in agricultural production; however, soil age (cultivating time) showed almost no influence on the distribution between SIC and SOC (Figure 5a). The relationship between soil spectra and the soil age was simulated using PLSR model, and a significant relationship can be found (Figure 5b), but the main PLS loading spectra showed the main related spectra range included most of the spectral ranges but excluded the range of 1,350–1,500 cm^−1^ (Figure 5c), which confirmed the result from distribution between PCA1 and PCA2. Therefore, the SIC content in surface layer of paddy soil was more likely linked with climatic change during the cultivating time.

Though there are some feedbacks from the soil C cycle to global warming, the feedback mechanisms are complicated [5], [34], [35]. Our findings can be the compensation for the feedbacks, which was indicated through the circular distribution between SIC and SOC in soil surface layer; most likely, the paddy soil can stock more inorganic C responding to global warming in soil surface layer, as a result, it may restrict the accumulation of SOC as well as the increase of global temperature.

### Conclusions

FTIR-PAS was a useful technique for investigating the spectroscopic profile of paddy soils at the surface of particles and aggregates. The spectral range of 1,350–1,500 cm^−1^ was found to best represent SOC and SIC signatures due to less interference. Soil organic carbon and SIC in the surface of soil samples studied were negatively correlated, suggesting that SIC may limit SOC content in soils. Additionally, the distribution between SIC and SOC in historic samples was related with climatic changes during cultivating times.
